# Understanding the association between pain and delirium in older hospital inpatients: systematic review and meta-analysis

**DOI:** 10.1093/ageing/afae073

**Published:** 2024-04-12

**Authors:** Nicola White, Juan Carlos Bazo-Alvarez, Michel Koopmans, Emily West, Elizabeth L Sampson

**Affiliations:** Marie Curie Palliative Care Research Department, Division of Psychiatry, Faculty of Brain Sciences, University College London, London, UK; Research Department of Primary Care and Population Health, University College London, London, UK; Escuela de Medicina, Universidad Cesar Vallejo, Trujillo, Peru; Respiratory Medicine Department, Erasmus Medical Centre in Rotterdam, Rotterdam, The Netherlands; e-Referrals Service, NHS England, Redditch, UK; Marie Curie Palliative Care Research Department, Division of Psychiatry, Faculty of Brain Sciences, University College London, London, UK; Department of Psychological Medicine, Royal London hospital, East London Foundation Trust, London, UK; Centre for Psychiatry and Mental Health, Queen Mary University London, London, UK

**Keywords:** pain, delirium, general hospitals, analgesia, older people, systematic review

## Abstract

**Objective:**

Delirium and pain are common in older adults admitted to hospital. The relationship between these is unclear, but clinically important. We aimed to systematically review the association between pain (at rest, movement, pain severity) and delirium in this population.

**Methods:**

PubMed, EMBASE, CINAHL, PsycINFO, Cochrane and Web of Science were searched (January 1982–November 2022) for Medical Subject Heading terms and synonyms (‘Pain’, ‘Analgesic’, ‘Delirium’). Study eligibility: (1) validated pain measure as exposure, (2) validated delirium tool as an outcome; participant eligibility: (1) medical or surgical (planned/unplanned) inpatients, (2) admission length ≥ 48 h and (3) median cohort age over 65 years. Study quality was assessed with the Newcastle Ottawa Scale. We collected/calculated odds ratios (ORs) for categorical data and standard mean differences (SMDs) for continuous data and conducted multi-level random-intercepts meta-regression models. This review was prospectively registered with PROSPERO [18/5/2020] (CRD42020181346).

**Results:**

Thirty studies were selected: 14 reported categorical data; 16 reported continuous data. Delirium prevalence ranged from 2.2 to 55%. In the multi-level analysis, pain at rest (OR 2.14; 95% confidence interval [CI] 1.39–3.30), movement (OR 1.30; 95% CI 0.66–2.56), pain categorised as ‘severe’ (OR 3.42; 95% CI 2.09–5.59) and increased pain severity when measured continuously (SMD 0.33; 95% CI 0.08–0.59) were associated with an increased delirium risk. There was substantial heterogeneity in both categorical (I^2^ = 0%–77%) and continuous analyses (I^2^ = 85%).

**Conclusion:**

An increase in pain was associated with a higher risk of developing delirium. Adequate pain management with appropriate analgesia may reduce incidence and severity of delirium.

## Key Points

- Pain and delirium commonly co-exist and are common in older people with unplanned acute hospital admissions.
- The causes of delirium are complex and multifactorial. Pain has been considered an important potential cause of delirium.
- Meta-analyses showed that pain was associated with increased risk of delirium.
- Data that controlled for analgesic medication suggests that pain is an independent risk factor for delirium.

## Introduction

Delirium is common in hospitalised older adults with a prevalence ranging from 29 to 64% [1], associated with worse outcomes including, higher mortality, longer inpatient stays and increased costs [2]. Identification and management of risk factors, (e.g. older age, dementia, visual/hearing impairment, illness severity) are key to prevention and treatment [3, 4]. The role of pain as a precipitant for delirium is less well understood, even though acute pain or worsening of chronic pain occurs in 38–84% of older people admitted to hospital [5]. Causal interactions between pain and delirium are complex and multidirectional, involving factors such as depression and sleep deprivation [6]. Although delirium can be caused by multiple stressors including infection, inflammation, drug toxicity and metabolic abnormalities, it is hypothesised that these all act through the final common pathway of acute cerebral stress [7]. Pain has a similar effect, acutely inducing catecholamine release and a short-term pro-inflammatory sympathetic response [8]. Chronic pain leads to dysfunction of the cortisol axis and prolonged over-activity of inflammatory cytokines. Therefore, acute or chronic pain may lower the threshold for delirium.

The core features of delirium (acute disturbance of attention, awareness and cognitive function with a fluctuating course) [9] lead to under recognition and under treatment of pain [6]. Hyperactive delirium, causes agitation, increased motor activity and restlessness, and may exacerbate pain by aggravating existing injuries or conditions [10]. Conversely, hypoactive delirium, where patients are withdrawn, lethargic and drowsy, impairs the ability to communicate pain [11]. Self-report is the gold standard for the assessment of pain, although in people with cognitive impairment such as dementia, observational tools are recommended. It is good practice to assess incident pain (at rest) and procedural pain (during movement) [12]. It could be hypothesised that pain at rest (which may be constant) is more likely to precipitate delirium than pain occurring during intermittent movement. It is also unclear how pain severity is associated with the occurrence of delirium.

Therefore, the aim was to systematically review and synthesise evidence on the association between pain and delirium in older adults admitted to hospital. Specific research objectives were to understand:

1. The association between pain (at rest and at movement) and delirium?
2. The association between pain severity and delirium risk?

## Methods

This systematic review is reported in accordance with the Preferred Reporting Items for Systematic reviews and Meta-Analyses guidance [13] and was prospectively registered with PROSPERO [18/5/2020] (CRD42020181346).

### Eligibility criteria

#### Inclusion criteria

Included studies reported (1) delirium (or synonyms) as a primary or secondary outcome, (2) pain was measured as an exposure, at least once during the baseline measurement or follow-up, (3) pain and delirium were measured using appropriate and validated tools. Studies were included if they used any validated delirium diagnostic criteria (DSM III, DSM IV and DSM V) or any validated tools including the Confusion Assessment Method (CAM) [14], Memorial Delirium Scale [15], Neecham Confusion Scale [16], Delirium Rating Scale [17]. For pain, all studies using self-reported (the gold-standard) or validated observational pain scales [12] were included. Study participants were: (1) medical or surgical (planned or unplanned) inpatients, (2) with an expected hospital length of stay of at least 48 h, (3) from cohorts with median age over 65 years old (if at least 80% of participants were aged above 60). We hypothesised that age would not be distributed normally at higher age ranges.

### Exclusion criteria

Studies were excluded if participants were admitted to intensive care units. This was because objectively measuring pain in this population, many of whom will be sedated, is challenging. We excluded studies published before January 1981, when the first operationalised criteria for delirium appeared in DSM-III, case reports, systematic reviews (after searching their reference lists), qualitative studies, opinion pieces, comments, conference abstracts, editorials and letters.

### Information sources

PubMed, EMBASE, CINAHL, PsycINFO, Cochrane and Web of Science on 19 April 2020, updated on 14 November 2022. References of published meta-analyses and included studies were searched for additional studies. References were exported into Endnote version X9.3.2 and de-duplicated.

### Search strategy

A preliminary PubMed search was completed using free text words and Medical Subject Headings for (pain AND/OR analgesic) AND delirium, using a wide range of synonyms, iteratively refined and translated for other databases (Supplementary Data S1). Studies of analgesic drugs were included if they reported data on pain and subsequent delirium.

### Selection process

An initial stage of screening titles and abstracts was conducted by two independent reviewers. Any study that was conducted in a hospital and included delirium and pain measures in the abstract or title underwent full text review. Two independent reviewers conducted full stage screening against all eligibility criteria. Disagreement was arbitrated by a third independent reviewer.

### Data collection process

Data were extracted from each paper by an individual reviewer (M.K., E.W., N.W.) checked by a second independent reviewer (M.K., E.W., N.W.) using Excel spreadsheet templates developed and piloted for this study. Disagreements were arbitrated by a third reviewer. All authors were emailed if full texts were not available, if study protocols were identified with no published result, or if the article described collection of data relevant to the review but did not present this. We contacted authors once and if no response, 1 month later.

### Data items

The outcome was the presence of delirium, and the main exposure was pain. The timepoints and frequency for pain and delirium assessments were extracted. Where categorical data were collected at multiple time points, the first assessment of pain and delirium was used for medical inpatients and the first day post-operatively for surgical patients. For continuous data, we extracted data given for all timepoints. Participant demographics (age, sex, diagnosis, admission reason), overall delirium prevalence delirium in the study population and study characteristics (study design, year of publication, country, setting, sample size) were extracted, where available.

### Study risk of bias assessment

Two reviewers (from M.K., E.W., N.W.) separately assessed articles using the Newcastle Ottawa Scale [18], comprising nine items (score range 0–9). Where there were discrepancies between independent raters, a third reviewer arbitrated (E.L.S.). Scores of 0–4, 5–7 and 8–9 are considered ‘Poor’, ‘Fair’ or ‘Good’ quality, respectively [18].

### Effect and synthesis measures

Study and patient demographics are summarised in Table 1. Measures used to record pain and delirium were described. Results are presented to reflect the review objectives. Pain data were analysed depending on their presentation within the studies, either:

1. Categorical pain data (pain described as either present or absent; pain severity described as mild, moderate, or severe as per standard cut-offs)
2. Continuous pain data (pain described by a numerical score with confidence range; an increase equating to more severe pain).

#### Categorical pain data synthesis measures

To report the association between categorical pain and delirium risk, unadjusted odds ratios (ORs) were extracted for the association between delirium (yes/no) and pain at movement (yes/no) and/or pain at rest (yes/no).

To report the association between severe pain (as a categorical variable), ‘severe’ pain was defined by studies if backed by a reference validating this cut-off point. Where this was not given, severe pain was defined as a Visual Analogue Scale (VAS) score of ≥7 and a verbal Rating Scale score of ≥3 [19].

For the data synthesis, when ORs were not given, an unadjusted OR was calculated using available information (e.g. from cross-tables). ORs were summarised with subgroup meta-analysis (random effects model) and a restricted maximum likelihood (REML) estimator, for each category of pain-defined subgroup. The heterogeneity I-squared was calculated, and an overall heterogeneity test performed, then stratified by pain category. The overall I-squared describes the percentage of the variability in mean effects from different subgroups that is due to genuine subgroup differences rather than sampling error. A test of group differences was performed to formally compare them.

#### Continuous pain data synthesis

Continuous pain scores were tabulated as means and standard deviation (SD), or median and inter quartile range (IQR), for people with and without delirium. Pain tools included in this analysis used different scales, therefore the standardised mean difference (SMD) was calculated between pain scores in those with and without delirium. SMDs were summarised using a random effects meta-analysis with a REML estimator, adjusted for the multiple timepoints within each study (Supplementary Data S2).

#### Adjusted multi-level meta-analyses

An additional mixed-effects multi-level meta-regression analysis was performed to adjust for those studies that reported more than one category of pain (i.e. same study and different category of pain available). For this analysis, the Higgins–Thompson I-squared was reported and should be interpreted similarly to the overall I-squared).

Some studies reported continuous data with different outputs that were included in the same meta-analysis. A multilevel random-intercepts meta-regression was performed to adjust for this, for which the random intercepts were the studies.

#### Data from studies that used controlled analyses

Studies including covariables in their analysis of pain and delirium risk were identified. It was not possible to complete a meta-analysis with the included studies due to heterogeneity of the controlling variables and different effect measures.

### Reporting bias assessment (sensitivity analyses)

Additional sensitivity analyses were conducted with continuous data, where there were missing data. One study did not report SD or SE [20], therefore SD values were imputed using the stratified averages of SD (by group). Additionally, three studies reporting median/IQR data [20–22] were transformed into mean/SD data [23]. Publication bias was evaluated using funnel plots for the main analysis only (Supplementary Data S3). Forest plots reported 95% confidence intervals and weights. All analyses were conducted using Stata MP 18.0 [24].

## Results

### Study selection

**Figure 1:**
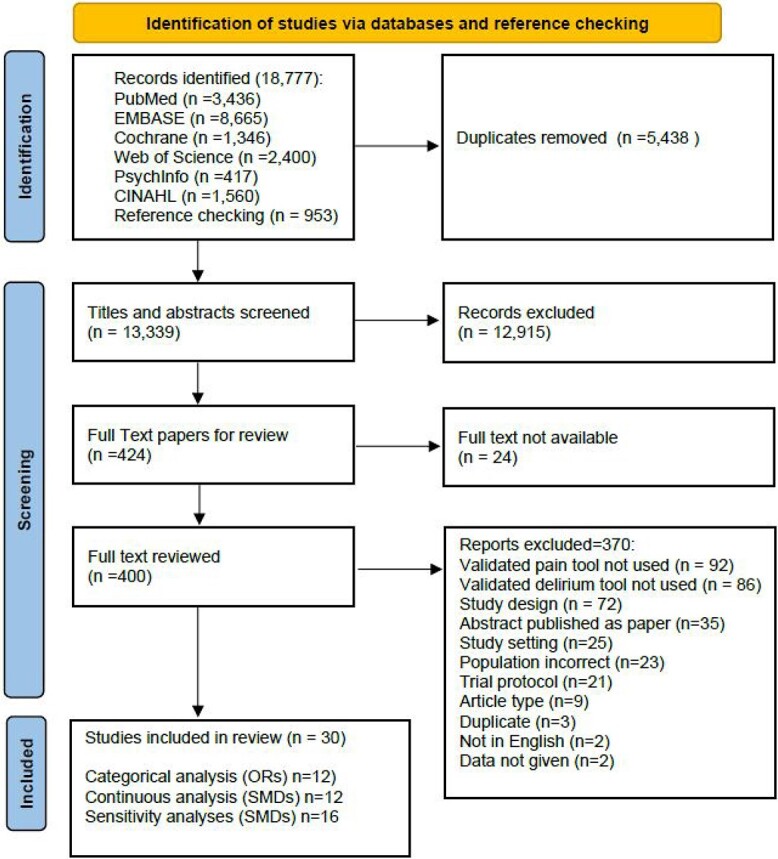
Study selection PRISMA diagram.

Searches identified a total of 18,777 papers. After duplicates were removed (5,438), we screened 13,339 papers on title and abstract, excluding 12,915. We reviewed 424 full text articles and 24 were excluded (full text copies not available). After full text review, 370 articles were excluded, most commonly for not using validated tools for pain (*n* = 92) or delirium (*n* = 86). A total of 30 studies were included (Figure 1).

### Study characteristics

Cohorts ranged from 43 to 2,168 participants undergoing a range of elective (hip/knee/shoulder, spinal, non-cardiac, neurosurgery), emergency (hip, general orthopaedic surgery) and mixed emergency and elective surgical cohorts (abdominal and urological, non-cardiac), and general medical admissions. Most studies were from the USA (*n* = 13, 43%) and were prospective cohorts (*n* = 20, 67%) (Table 1).

**Table 1:** Population and study characteristics

**First author**	**Year**	**Setting**	***N***	**Study type**	**Population**	**Reason for admission**	**Quality**
Alvarez-Bastidas [36]	2018	Mexico	100	Cross sectional	Adults (60 +)	Undergoing surgery less than 60 min and receiving analgesia	Fair
Ansaloni [46]	2020	Italy	357	Case–control	Adults (65+)	Emergency or elective operations	Fair
Bjoro [31]	2008	Norway	204	Cohort prospective	Adults (65+)	Hip fracture surgery	Good
Bowman [20]	1997	USA	43	Cohort prospective	Adults	Undergoing orthopaedic hip surgery	Fair
Brown [47]	2016	USA	89	Cohort prospective	Adults (70+)	Elective spine surgery	Fair
Contín [48]	2005	Spain	236	Cohort prospective	Adults (51 to 80)	Elective orthopaedic surgery	Fair
Duprey [21]	2022	USA	560	Secondary analysis	Adults (70+)	Undergoing major elective surgery	Good
Feast [32]	2018	UK	230	Cross sectional	Dementia Adults (70+)	Unplanned medical hospital admission	Good
Jain [49]	2011	USA	400	RCT Secondary analysis	Adults (65+) or < 65 if history of delirium	Elective total hip or knee replacement surgery	Poor
Johansson [50]	2013	Sweden	49	Cohort prospective	Adults (70+)	Hip fracture surgery	Fair
Kosar [38]	2014	USA	459	Cohort prospective	Adults (70+)	Major elective surgery	Good
Kubota [51]	2018	Japan	2,168	Cohort retrospective	Adults	Surgical ward admission	Good
Leung [52]	2009	USA	335	Nested cohort prospective	Adults (65+)	Noncardiac surgery	Fair
Leung [53]	2013	USA	581	Cohort prospective	Adults (65+)	Scheduled for major non-cardiac surgery	Good
Lewis [29]	2017	Tanzania	494	Cross sectional	Adults (60+)	Admitted to medical wards	Good
Li [54]	2019	China	111	Cohort prospective	Adults (65+)	Acute STEMI following primary PCI	Fair
Liang [55]	2014	Taiwan	232	Cohort prospective	Adults (60+)	Orthopaedic surgery	Good
Lin [33]	2016	Taiwan	1,609	Cohort prospective	Adults (60+)	Elective noncardiac surgery and general anaesthesia	Good
Liu [22]	2022	China	184	Cohort prospective	Adults (60+)	Thoracic and abdominal surgery	Good
Lynch [56]	1998	USA	361	Cohort prospective	Adults (50+)	Major elective noncardiac operations	Good
Matsuo [45]	2017	Japan	207	Cohort prospective	Adults (20+)	Advanced cancer patients receiving corticosteroids to treat fatigue or anorexia	Fair
Morrison [34]	2003	USA	539	Cohort prospective	Adults	Hip fracture	Fair
Narayanan [39]	2022	India	50	Cohort prospective	Adults (61+)	Elective onco-surgery	Good
Oh [35]	2008	Korea	224	Cohort retrospective	Adults (71+)	Neurosurgical operation	Good
Roche-Albero [57]	2021	Spain	133	Cohort prospective	Adults (65+)	Osteoporotic hip fracture	Fair
Salottolo [58]	2022	USA	517	Cohort prospective	Adults (55–90)	Traumatic hip fracture	Good
Sieber [59]	2011	USA	236	Cohort prospective	Adults (65+)	Hip fracture repair	Fair
Susano [60]	2019	USA	715	Cohort retrospective	Adults (65+)	Spinal surgery	Good
Vaurio [30]	2006	USA	331	Cohort prospective	Adults (65+)	Elective noncardiac surgery	Fair
Xue [61]	2016	China	358	Cohort prospective	Adults (65+)	Transurethral resection of prostate	Fair

### Risk of bias

Fifteen studies were rated ‘Good’ for quality (50%), 14 ‘Fair’ (47%) and 1 study ‘Poor’ (3%). We identified seven primary and one secondary analysis of randomised controlled trials.

### Results of studies

#### Measures reported

##### Pain

Out of 30 included studies, 26 (87%) collected data on pain using a Numerical Rating (NRS) or VAS scale [12]. Other pain scales used were the PAINAD [25], pain subscale of the Support Team Assessment Schedule—Japanese [26], checklist of non-verbal pain indicators[27] and brief pain inventory [28] (details on measures and frequency of assessment -Supplementary Data S2).

##### Delirium

Diagnostic tools and delirium prevalence are given in Supplementary Data S3. Most studies (*n* = 24, 80%) used the CAM [14] to diagnose delirium: prevalence ranged from 2.2 to 55.0% (mean 22.7%).

### Results of synthesis

#### What is the association between pain (at rest and at movement) and delirium risk?

Studies reporting pain at rest and movement only used categorical pain. In total, 14 studies analysed categorical pain data, 12 of which presented data suitable to include in the meta-analysis.

**Figure 2:**
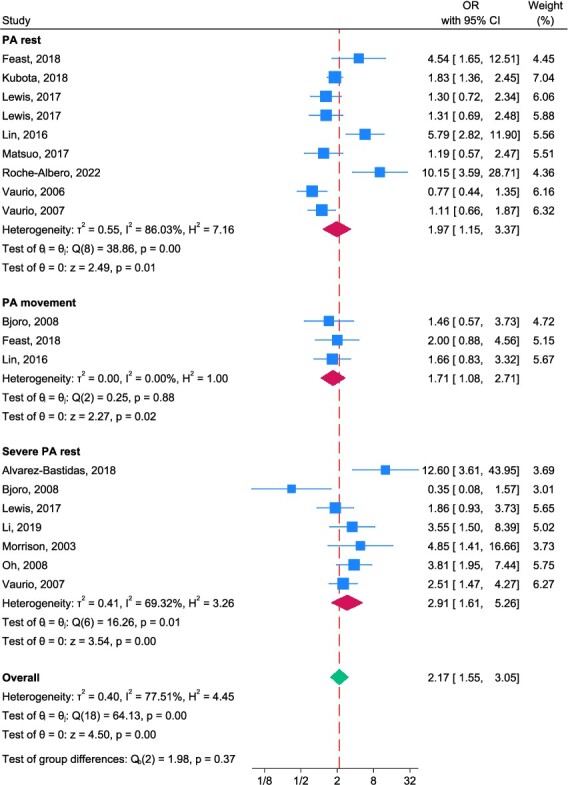
Forest plot for subgroup meta-analysis, summarising ORs for the unadjusted association between pain and delirium risk (categorical pain data). Note: where studies are included more than once, data are derived from the same group of patients, but from multiple different time points; a higher OR value means a higher risk of delirium.

Figure 2 summarises the OR for pain (categorical) and delirium risk, with subgroups of pain at rest and at movement. Overall, patients experiencing pain were 2.17 times more likely to experience delirium (95% CI 1.35–3.06).

##### Pain at rest

Six studies with nine datasets were included in the meta-analysis. Two studies reported pain at rest using the VAS (grouping responses of 1–6 [29] or 1–4 [30] against higher scores) as well as a categorical response to pain at rest (Yes/No, which dichotomised VAS responses to 0 (no pain) versus other scores [pain]). Patients with pain at rest were 1.97 times (95% CI 1.15–3.37) more likely to have delirium.

##### Pain at movement

Three studies reported data on pain at movement and delirium [31–33]. Two studies dichotomised a NRS at 3 [31, 33]. Feast *et al.* [32] reported on the presence of pain at movement with the PAINAD tool [25]. Patients experiencing pain at movement were 1.71 times more likely to experience delirium (95% CI 1.08–2.71).

The test for group differences (Qb(2) = 1.98; *P* = 0.37) of all categorical data indicates that these findings are just a trend, although the overall I-squared indicates that 77.5% variability in mean effects from different subgroups is due to genuine subgroup differences.

#### Is there an association between pain severity and delirium risk?

For the second review objective, both categorical and continuous data were available. Seven studies specifically reported a ‘severe’ pain category but used different cut-offs: ≥3 [31], ≥4 [33, 34], ≥5 [30], >6.8, [35] ≥7 [36, 37]. Figure 2 summarises the OR for severe pain at rest (categorical) and delirium risk. No datapoints were reported for severe pain at movement (categorical) and delirium risk. Patients experiencing severe pain at rest were 2.91 times more likely to experience delirium (95% CI 1.61–5.26). As indicated previously, this finding in the test for group differences was only a trend (Qb(2) = 1.98; *P* = 0.37).

**Figure 3:**
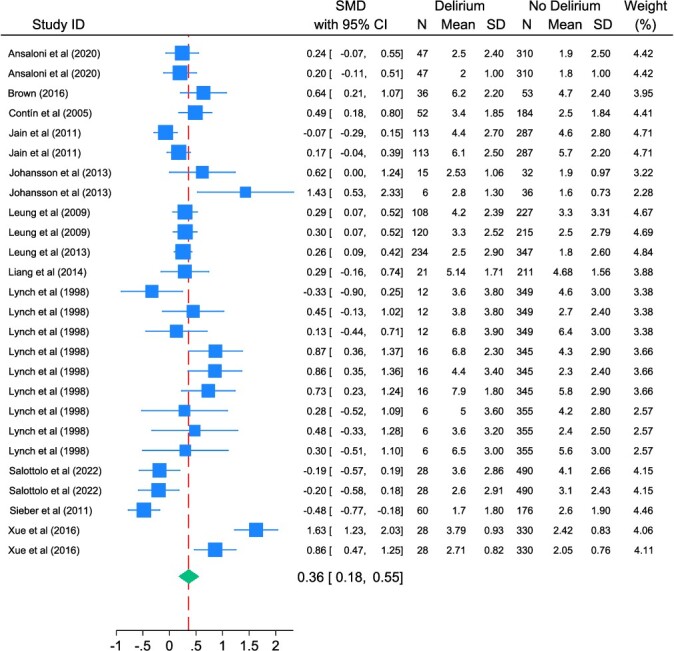
Forest plot for meta-analysis summarising SMDs between people with and without delirium (using continuous pain data). Note: where studies are included more than once, data are derived from the same group of patients, but from multiple different time points; a higher SMD value means a higher risk of delirium.

In total, 16 studies reported continuous pain scores. Four studies were included only in the sensitivity analysis as they reported data other than means or SDs. The remaining 12 studies indicated that, overall, patients who had delirium reported a standardised mean pain score of 0.36 (95% CI 0.18–0.55) higher than those without delirium. However, there was substantial study heterogeneity (I^2^ = 85%; Q = X2(25) = 130.60; *P* < 0.001) (Figure 3). This suggests patients who had delirium report, on average, 12% more pain than patients who did not have delirium (SMD 0.36 * 34.1 = 12.28%).

#### Adjusted multi-level analyses

The trend between delirium and pain (reported as categorical data) was consistent in multi-level meta-analysis: pain at rest (OR = 2.14; 95% CI 1.39–3.30), pain at movement (OR = 1.30; 95% CI 0.66–2.56) and severe pain at rest (OR = 3.42; 95% CI 2.09–5.59). There was significant heterogeneity among the subgroups (Higgins–Thompson I-squared = 75.6%).

In multi-level meta-analysis of continuous data, the trend between pain and delirium risk was consistent. The pain scores were higher for those with delirium (standardised pain score difference = 0.33; 95% CI 0.08–0.59); however, there was considerable study heterogeneity (Higgins–Thompson I-squared = 85.3%; Qm = X2(25) = 130.60; *P* < 0.001).

#### Controlled analyses

Three studies [30, 34, 35] examined the association between pain and delirium, adjusting for a range of factors including analgesics [30, 34] (Supplementary Data S6). All showed significant association between severe pain and delirium: Morrison [34] RR 9, 95% CI 1.8–45.2; Oh [35] OR 1.99, 1.45, 4.16 and Vaurio [30] OR 3.72, 95% CI 1.54–8.96.

### Data not included in a meta-analyses

Two studies reported data unsuitable for meta-analysis [38, 39] (Supplementary Data S7). Kosar *et al.* [38] reported delirium risk increased with pain severity, particularly for those experiencing severe current pain. Narayanan *et al.* [39] found no association between pain and delirium.

### Reporting bias

The initial sensitivity analysis (with imputed values) found the standardised mean pain score for those with delirium was 0.46 higher than those without (95% CI 0.28–0.64) with a high level of heterogeneity (Higgins–Thompson I-squared = 84.7%; Qm = X2(31) = 156.74; *P* < 0.001). In multi-level meta-analysis, the delirious group reported a higher pain score (standardised pain score difference = 0.39; 95% CI 0.13–0.64; *P* = 0.003), heterogeneity was considerable (I^2^ = 80%).

In the second sensitivity analysis (with imputed values and transformed medians), the standardised mean pain score for those with delirium was 0.49 higher than those without (95% CI 0.34–0.64). In the multi-level meta-analysis, those with delirium reported higher pain scores (standardised pain score difference = 0.42; 95% CI 0.21–0.63; *P* < 0.001), heterogeneity was considerable (I^2^ = 80%). For further information on interpreting SMD scores, see Supplementary Data S4.

#### Publication bias

Funnel plots for continuous and categorical analyses contain few studies but show a relatively symmetrical pattern (Supplementary Data S5).

## Discussion

### Key findings

Understanding modifiable risk factors for delirium is vital to improve delirium prevention and care, but there has been little systematic exploration of the association between pain and delirium. In this review, patients with pain at rest and at movement were almost twice as likely to develop delirium (OR 2.14; 95% CI 1.39–3.30 and OR 1.30; 95% CI 0.66–2.56, respectively). In addition, patients with ‘severe’ pain were over three times more likely to develop delirium (OR 3.42; 95% CI 2.09–5.59). Analysis of pain as a continuous variable demonstrated increasing delirium risk with increasing pain (SMD 0.33; 95% CI 0.08–0.59), suggesting that patients with delirium reported an average pain score of 12% higher than those without delirium.

Pain at rest appears to have a greater effect on delirium occurrence. This finding supports previous evidence; pain at rest is often consistent and thus more likely to lead to cerebral stress, possibly mediated through the sympathetic nervous system [8], or disrupted sleep patterns [6], than pain which occurs intermittently on movement. Our findings suggest a possible dose–response association between increasing pain and increasing delirium risk. Further research exploring causality of this association is needed.

The association between pain and delirium may be confounded by other factors. Most included studies (27/30) were of surgical cohorts, undergoing procedures such as hip arthroplasty or abdominal surgery. Many participants would have received opioid analgesics or benzodiazepines, which independently increase delirium risk [40]. There was little mention of other factors important in pain and delirium care such as managing sleep quality, optimising the environment, polypharmacy etc. However, analyses that controlled for analgesic use, particularly opioids, demonstrated significant associations between pain and delirium [30, 34, 35].

The increased delirium risk associated with increased pain may be due to use of higher opioid doses with more severe pain. Opioids are thought to commonly precipitate delirium in older people [40] but evidence is inconsistent. Other meta-analyses have shown a lack of significant association between opioids and delirium [41]. There is evidence of a dose—response relationship particularly when >90 mg morphine equivalent daily is given [41] but, also data which suggests that, particularly in acute severe pain such as hip fracture, lower opioid doses (suggesting undertreated pain) may increase delirium risk [40].

### Comparison with other literature

The prevalence of delirium in studies selected for this review reflects the wide variation found throughout the literature [1].To our knowledge there are no other systematic reviews that have specifically examined the association between pain and delirium in this population. Reviews and meta-analyses in ICU populations of multicomponent delirium reduction bundles (which include rigorous pain management) demonstrate reduced delirium rates, but it is not possible to disaggregate the impact of the individual pain management component [42]. One systematic review of risk factors for delirium in older adults in the emergency department reported that severe pain, rather than opioid use was associated with delirium [43] and one systematic review in specialist palliative care patients found inconclusive evidence that pain is associated with delirium [44].

### Strengths and limitations

This was a robust and comprehensive review. There was considerable study heterogeneity with differing populations, a wide range of reported delirium prevalence (2.2–55.0%), use of a variety of cut-off scores for pain severity and pain scales. This was managed by standardising continuous pain scores where scales used different ranges, but the method is imperfect. Some studies omitted SD/SE data or only reported median and IQR. A sensitivity analysis was conducted with imputed values and transformed medians, but this did not alter findings of an association between increasing pain scores and delirium.

Many selected studies were not designed or powered to answer our study question, this is important to ensure adequate data on potential confounders of the pain-delirium relationship are considered, particularly opioids and benzodiazepines. It is challenging to measure pain in people with delirium, which may impair ability to understand and communicate [6], thus pain prevalence in included studies may have been underestimated. There was variability in the timepoints described in the studies. Therefore, it is challenging to explore temporal relationships. There are also inconsistencies with how studies used continuous pain scales, for example some used VAS scores ranged from 0 to 10, whilst others used VAS anchored from 0 to 8. Similarly, studies were inconsistent in how they defined ‘severe’ pain categories. Pain and delirium can both fluctuate and may be missed if observations are made infrequently. Only three studies [32, 37, 45] studied non-surgical populations.

### Implications for research

Further studies including a broader range of older acute hospital inpatients, particularly non-surgical, or those who live in the community such as care home residents are needed. Future studies require repeated measurements of pain and delirium to explore temporal associations, standardised pain tools valid in patients who are unable to communicate, for example those with dementia, and must consider important confounding factors such as analgesic use and illness severity.

### Clinical implications

Pain and delirium management are fundamental components of care for older people. Although there are concerns that opioid analgesics may increase delirium risk; these data suggest that pain is an important driver of delirium in older acute hospital inpatients. Adequate pain management may therefore reduce the incidence and severity of delirium, carefully balanced with the side effects of analgesic medications.

## Supplementary Material

aa-23-1955-File002_afae073

aa-23-1955-File003_afae073

## Data Availability

Template data collection forms; data extracted from included studies; data used for all analyses; analytic code and other materials used in the review can be obtained from the authors.
