# Supplementary material for: Understanding the association between pain and delirium in older hospital inpatients: systematic review and meta-analysis
Source: Age Ageing. 2024 Apr 12;53(4):afae073. doi: 10.1093/ageing/afae073 (PMC11014791; doi:10.1093/ageing/afae073)
Supplement: aa-23-1955-File002_afae073 [file aa-23-1955-file002_afae073.docx]

**Supplementary Data 1- Search strategies**

**PubMed**

#1 Pain[mh]

#2 Pain*

#3 Pain measurement[Mh]

#4 Pain clinics[Mh]

#5 Analgesia[Mh]

#6 Analgesics[Mh]

#7 Hyperalgesia[Mh]

#8 Hyperesthesia[Mh]

#9 Analgesi*

#10 Hyperalgesi*

#11 Hyperesthesi*

#12 Dyscomfort*

#13 "Visual analogue scale"[TIAB] OR VAS[TIAB] OR "Numeric rating scale"[TIAB] OR NRS[TIAB]

#14 OR/1-13

#15 Delirium[mh]

#16 Deliri*

#17 Confusion[Mh]

#18 Confusi*

#19 "Acute confusional state"

#20 acute confusion*

#21 "Acute brain faillure"

#22 "acute brain syndrome"

#23 "metabolic encephalopathy"

#24 "acute psycho‐organic syndrome"

#25 "clouding of consciousness"

#26 "exogenous psychosis"

#27 "toxic psychosis"

#28 "toxic confusion"

#29 "acute cerebral insufficiency"

#30 agitat*

#31 OR/15-30

#32 Aged[Mh]

#33 ("Aged") AND (((((65-years-and) OR 65-and) OR Old) OR High) OR Above)

#34 "Elderly"[TIAB]

#35 "Old* people"

#36 "Old* Person*"

#37 High-age*

#38 65-years-and

#39 above-65

#40 Older[TIAB]

#41 Senior*[TIAB]

#42 OR/32-41

#43 #14 AND #32 AND #42

**EMBASE**

exp pain measurement/

3 exp pain clinic/

4 Analgesia/

5 analgesics/

6 hyperalgesia/

7 hyperesthesia/

8 pain*.mp. [mp=title, abstract, heading word, drug trade name, original title, device manufacturer, drug manufacturer, device trade name, keyword, floating subheading word, candidate term word]

9 analgesi*.mp. [mp=title, abstract, heading word, drug trade name, original title, device manufacturer, drug manufacturer, device trade name, keyword, floating subheading word, candidate term word]

10 hyperalgesi*.mp. [mp=title, abstract, heading word, drug trade name, original title, device manufacturer, drug manufacturer, device trade name, keyword, floating subheading word, candidate term word]

11 Hyperesthesi*.mp. [mp=title, abstract, heading word, drug trade name, original title, device manufacturer, drug manufacturer, device trade name, keyword, floating subheading word, candidate term word]

12 Discomfort*.mp. [mp=title, abstract, heading word, drug trade name, original title, device manufacturer, drug manufacturer, device trade name, keyword, floating subheading word, candidate term word]

13 "VAS".ti,ab.

14 "Visual-analogue-scale".ti,ab.

15 NRS.ti,ab.

16 "Numeric-analogue-scale".ti,ab.

17 1 or 2 or 3 or 6 or 7 or 8 or 10 or 11 or 12 or 13 or 14 or 15 or 16

18 4 or 5 or 9 or 17

19 exp delirium/

20 exp confusion/

21 Deliri*.mp. [mp=title, abstract, heading word, drug trade name, original title, device manufacturer, drug manufacturer, device trade name, keyword, floating subheading word, candidate term word]

22 confusi*.mp. [mp=title, abstract, heading word, drug trade name, original title, device manufacturer, drug manufacturer, device trade name, keyword, floating subheading word, candidate term word]

23 (acute adj confusional adj state).mp. [mp=title, abstract, heading word, drug trade name, original title, device manufacturer, drug manufacturer, device trade name, keyword, floating subheading word, candidate term word]

24 (acute adj brain adj failure).mp. [mp=title, abstract, heading word, drug trade name, original title, device manufacturer, drug manufacturer, device trade name, keyword, floating subheading word, candidate term word]

25 (acute adj brain adj syndrome).mp. [mp=title, abstract, heading word, drug trade name, original title, device manufacturer, drug manufacturer, device trade name, keyword, floating subheading word, candidate term word]

26 (metabolic adj encephalopathy).mp. [mp=title, abstract, heading word, drug trade name, original title, device manufacturer, drug manufacturer, device trade name, keyword, floating subheading word, candidate term word]

27 (acute adj psycho-organic adj syndrome).mp. [mp=title, abstract, heading word, drug trade name, original title, device manufacturer, drug manufacturer, device trade name, keyword, floating subheading word, candidate term word]

28 (clouding adj of adj consciousness).mp. [mp=title, abstract, heading word, drug trade name, original title, device manufacturer, drug manufacturer, device trade name, keyword, floating subheading word, candidate term word]

29 (exogenous adj psychosis).mp. [mp=title, abstract, heading word, drug trade name, original title, device manufacturer, drug manufacturer, device trade name, keyword, floating subheading word, candidate term word]

30 (toxic adj psychosis).mp. [mp=title, abstract, heading word, drug trade name, original title, device manufacturer, drug manufacturer, device trade name, keyword, floating subheading word, candidate term word]

31 (toxic adj confusion).mp. [mp=title, abstract, heading word, drug trade name, original title, device manufacturer, drug manufacturer, device trade name, keyword, floating subheading word, candidate term word]

32 (acute adj cerebral adj insufficiency).mp. [mp=title, abstract, heading word, drug trade name, original title, device manufacturer, drug manufacturer, device trade name, keyword, floating subheading word, candidate term word]

33 Agitat*.mp. [mp=title, abstract, heading word, drug trade name, original title, device manufacturer, drug manufacturer, device trade name, keyword, floating subheading word, candidate term word]

34 19 or 20 or 21 or 22 or 23 or 24 or 25 or 26 or 27 or 28 or 29 or 30 or 31 or 32 or 33

35 exp aged/

36 (aged adj2 ("65-years-and" or old or high or above or "65 and")).mp.

37 (old* adj people).mp. [mp=title, abstract, heading word, drug trade name, original title, device manufacturer, drug manufacturer, device trade name, keyword, floating subheading word, candidate term word]

38 (old* adj Person*).mp. [mp=title, abstract, heading word, drug trade name, original title, device manufacturer, drug manufacturer, device trade name, keyword, floating subheading word, candidate term word]

39 "High-age*".mp. [mp=title, abstract, heading word, drug trade name, original title, device manufacturer, drug manufacturer, device trade name, keyword, floating subheading word, candidate term word]

40 "65 years and".mp. [mp=title, abstract, heading word, drug trade name, original title, device manufacturer, drug manufacturer, device trade name, keyword, floating subheading word, candidate term word]

41 "above 65".mp. [mp=title, abstract, heading word, drug trade name, original title, device manufacturer, drug manufacturer, device trade name, keyword, floating subheading word, candidate term word]

42 Older.ti,ab.

43 elderly.ti,ab.

44 senior*.ti,ab.

45 35 or 36 or 37 or 38 or 39 or 40 or 41 or 42 or 44 or 43

46 18 and 34 and 45

**Search strategy CINAHL database**

S60 S21 AND S37 AND S59

S59 S50 OR S58

S58 S51 AND S57

S57 S52 OR S53 OR S54 OR S55 OR S56

S56 above

S55 high

S54 old

S53 "65 and"

S52 "65 years and"

S51 aged

S50 S38 OR S39 OR S40 OR S41 OR S42 OR S43 OR S44 OR S45 OR S46 OR S47 OR S48 OR S49

S49 AB older

S48 AB senior

S47 TI senior

S46 TI Older

S45 "above 65"

S44 "65-years-and"

S43 "High age*"

S42 "Old* person*"

S41 "Old* people"

S40 AB elderly

S39 TI elderly

S38 (MH "Aged+")

S37 S22 OR S23 OR S24 OR S25 OR S26 OR S27 OR S28 OR S29 OR S30 OR S31 OR S32 OR S33 OR S34 OR S35 OR S36

S36 agitat*

S35 "acute cerebral insufficiency"

S34 "toxic confusion"

S33 "toxic psychosis"

S32 "exogenous psychosis"

S31 "clouding of consicousness"

S30 "acute psycho-organic syndrome"

S29 "metabolic encephalopathy"

S28 "acute brain syndrome"

S27 "acute brain failure"

S26 "acute confusional state"

S25 confusi*

S24 Deliri*

S23 (MH "Confusion+")

S22 (MM "Delirium")

S21 S1 OR S2 OR S3 OR S4 OR S5 OR S6 OR S7 OR S8 OR S9 OR S10 OR S11 OR S12 OR S13 OR S14 OR S15 OR S16 OR S17 OR S18 OR S19 OR S20

S20 TI "NRS"

S19 AB "NRS"

S18 AB "numeric analogue scale"

S17 TI "numeric analogue scale"

S16 TI "VAS"

S15 AB "VAS"

S14 AB "visual analogue scale"

S13 TI "visual analogue scale"

S12 discomfort*

S11 Hyperesthesi*

S10 hyperalgesi*

S9 analgesi*

S8 pain*

S7 (MM "Hyperalgesia")

S6 (MM "Hyperesthesia")

S5 (MH "Analgesia+")

S4 (MH "Analgesics+")

S3 (MM "Pain Clinics")

S2 (MM "Pain Measurement")

S1 (MH "Pain+")

**Search strategy PsycINFO database**

S56 S18 AND S34 AND S55

S55 S35 OR S36 OR S37 OR S38 OR S39 OR S40 OR S41 OR S42 OR S43 OR S44 OR S45 OR S46 OR S54

S54 S47 AND S53

S53 S48 OR S49 OR S50 OR S51 OR S52

S52 above

S51 high

S50 old

S49 "65 and"

S48 "65 years and"

S47 aged

S46 AB older

S45 AB senior

S44 TI senior

S43 TI Older

S42 "above 65"

S41 "65-years-and"

S40 "High age*"

S39 "Old* person*"

S38 "Old* people"

S37 AB elderly

S36 TI elderly

S35 DE "Geriatrics" OR DE "Geriatric Assessment" OR DE "Geriatric Psychiatry" OR MM "Gerontology"

S34 S19 OR S20 OR S21 OR S22 OR S23 OR S24 OR S25 OR S26 OR S27 OR S28 OR S29 OR S30 OR S31 OR S32 OR S33

S33 agitat*

S32 "acute cerebral insufficiency"

S31 "toxic confusion"

S30 "toxic psychosis"

S29 "exogenous psychosis"

S28 "clouding of consciousness"

S27 "Acute psycho-organic syndrome"

S26 "metabolic encephalopathy"

S25 "acute brain syndrome"

S24 "acute brain failure"

S23 "Acute confusional state"

S22 confusi*

S21 deliri*

S20 MM "Mental Confusion"

S19 MM "Delirium"

S18 S1 OR S2 OR S3 OR S4 OR S5 OR S6 OR S7 OR S8 OR S9 OR S10 OR S11 OR S12 OR S13 OR S14 OR S15 OR S16 OR S17

S17 TI "NRS"

S16 AB "NRS"

S15 AB "Numeric analogue scale"

S14 TI "Numeric analogue scale"

S13 TI "Numeric analogue scale"

S12 TI "visual analogue scale"

S11 AB "visual analogue scale"

S10 AB "VAS"

S9 TI "VAS"

S8 dyscomfort*

S7 Hyperalgesi*

S6 hyperesthesi*

S5 analgesi*

S4 pain*

S3 MM "Analgesia"

S2 DE "Pain" OR DE "Aphagia" OR DE "Back Pain" OR DE "Chronic Pain" OR DE "Headache" OR DE "Myofascial Pain" OR DE "Neuralgia" OR DE "Neuropathic Pain" OR DE "Somatoform Pain Disorder"

S1 MM "Pain Measurement"

Supplementary Data 2: Pain data

| **First Author** | **Pain measure** | **Pain measure score range** | **Score interpretation** | **Method of analysis** | **Frequency** | **Timepoints** |
| --- | --- | --- | --- | --- | --- | --- |
| Alvarez-Bastidas (2018) [25] | VAS | nr | > 7 severe pain. | Categorical | Once | post-surgery |
| Ansaloni (2020) [26] | VAS & Simple verbal scale | nr | Increase in score = worse pain | Continuous | Four | Preop, 3 consecutive days postop (only data pre-op is reported) |
| Bjoro (2008) [27] | CNPI change score (presence) | 6 domains | 0 to 6 for each position (0 to 12); no change between scores at rest and movement "Normal range"; change between rest and movement >0 = "Increased risk" | Categorical | Four days, at rest and movement | Baseline (day of surgery) 3 consecutive days post-operative |
|  | VRS (intensity) | 5-point descriptor: no pain, mild pain, moderate pain, severe pain, and very severe | 0-3 "Normal", 4-5 "Increased risk" |  |  |  |
| Bowman (1997) [20] | VAS | 10cm length | Increase in score = worse pain | Continuous ± | Eleven | Admission, then twice a day for 5 days |
| Brown (2016) [28] | NRS | 0-10 | Increase in score = worse pain | Continuous | unclear | Unclear |
| Contín (2005) [29] | VAS | 1 to 8 | Increase in score = worse pain | Continuous | multiple | Day before surgery, 7 days post-surgery |
| Duprey (2022) [21] | BPI | nr | nr | Continuous ± | multiple | During hospitalisation, worst pain and average pain. |
| Feast (2018) [30] | PAINAD* | Five domains | Each domain scores 0-2; maximum score 10. ≥ 2 indicates pain. | Categorical | multiple | Admission; every 4 (±1) days until discharge; at movement and at rest |
| Jain et al (2011) [31] | NRS | 0 (no pain) to 10 (worst pain imaginable) | Increase in score = worse pain | Continuous | unclear; results report two | Day of surgery, post-operative day 1 |
| Johansson (2013) [32] | Verbal description scale | 1 (no physical pain) to 5 (unbearable physical pain) | Increase in score = worse pain | Continuous | Four times, at rest and movement | Pre-op, Day 1 post, day 3 post, day before discharge |
| Kosar (2014) [33] | BPI (3 items) | 0 (no pain) to 10 (worst pain imaginable); current pain, average pain, and worst pain in the past 7 days. | 0 = no pain; 1-3 = mild; 4-6 = moderate; 7-10 = severe | Categorical† | One | Pre-operative |
| Kubota (2018) [34] | VAS | nr | 5+ = "positive" 4- = "negative" | Categorical | Unclear | Unclear |
| Leung (2013) [36] | NRS | 0 (no pain) 10 (worst imaginable pain) | ≤4=mild; ≥ 5=severe | Continuous | Four | Pre- and post- days 1 and 2; paper reports post-op data only as pre-op non-significant |
| Leung 2009 [35] | verbal VAS | 0 (no pain) to 10 (maximum pain) | Increase in score = worse pain | Continuous | Three | Pre-operative, post op day 1 and 2. |
| Lewis (2017) [56] | VAS/PAINAD* | 0 (no pain) to 10 (Severe pain). | None 0, Mild 1-2, Moderate 3-6, Severe 7-10. | Categorical | One | Within 24 hours of admission |
| Li (2019) [38] | VAS | nr | >4 = presence of pain | Categorical | One | Post-operative current pain |
| Liang (2014) [39] | VAS | nr | nr | Continuous | One | Pre-operative |
| Lin (2016) [40] | VNRS | 0 (no pain) to 10 (worst imaginable pain) | ≤3 adequate pain relief, >3 inadequate pain relief | Categorical | Three | Post-operative day 1-3 |
| Liu (2022) [22] | VAS | nr | nr | Continuous ± | Two | Pre- and post-operative |
| Lynch (1998) [41] | VAS | 0 (no pain) to 10 (the worst pain imaginable). | Increase in score = worse pain | Continuous | Three | Post-operative 3 days. |
| Matsuo (2017) [42] | STAS-J item on pain | 5-point scale: 0 (None) 4 (Severe and continuous overwhelming symptom(s). Unable to think of other matters). | Increase in score = worse pain | Categorical | One | Baseline assessment |
| Morrison (2003) [43] | Verbal description | 1 (no pain) to 5 (very severe) | 1 to 3 - no risk factor; 4 to 5 - risk factor | Categorical | Multiple | Pain at rest. Pain was indicated as a risk factor if scored 4/5 within 48 hours of delirium. Pain recorded daily up to post op day 3 for patients without delirium. |
| Narayanan (2022)[44] | NRS | 0 (no pain) 10 (worst pain imaginable) | 0 = No pain; 1-3 = mild; 4-7 = moderate; 8-10 = severe | Categorical† | Four | Pain at rest and dynamic pain. Pre-op (once) and post-op three days (twice a day). Study doesn't report baseline pain. |
| Oh (2008) [45] | VAS | 10cm line | Dichotomized at 6.8. | Categorical | Not described | Postoperative |
| Roche-Albero (2021) [46] | VAS | nr | ≥ 6 | Categorical | Two | Pre and pot operative |
| Salottolo (2022) [47] | NRS | 0 (no pain) 10 (worst imaginable pain) | Increase in score = worse pain | Continuous | Five | Arrival, admission, pre-op, post-op, discharge. |
| Sieber (2011) [48] | NRS | 0 (no pain) 10 (worst pain imaginable) | Increase in score = worse pain | Continuous | Multiple | PACU pain - pain on discharge from PACU; pain on operative day = average pain since discharge from PACU. Pain scores represent average pain for previous 24 hours. On PACU routinely assessed. Assessed every 4 hours after discharged from PACU. |
| Susano (2019) [49] | NRS | 0 to 10 | Increase in score = worse pain | Continuous ± | One | Postoperative mean pain score day 1 |
| Vaurio (2006) [50] | VAS | 0 (no pain), 1-4 (moderate), 5-10 (severe). | None, moderate, severe. | Categorical | Three | Pre-op <48hrs before surgery, day 1 and day 2 post-op |
| Xue (2016) [51] | VAS | "No pain" to "very severe pain". | Increase in score = worse pain | Continuous | Three | Pre- and post- op days 1 & day 2 at rest. |

VRS verbal rating scale, VAS visual analogue scale, NRS numerical rating scale, STAS-J Support Team Assessment Schedule - Japanese, PAINAD Pain Assessment IN Advanced Dementia, VNRS verbal numerical rating scale, BPI brief pain inventory, CNPI checklist of non-verbal pain indicators, nr not reported, † Not included in meta-analysis as data not reported, ± data for standard deviation not reported so calculated and included in a sensitivity analysis.

Supplementary Data 3: Delirium data

| **First Author** | **Delirium tool** | **Prevalence (%)** | **Frequency measured** | **Timepoint** |
| --- | --- | --- | --- | --- |
| Alvarez-Bastidas (2018) | CAM | 18 | Once | Post-surgery |
| Ansaloni (2020) | CAM | 13.2 | Six | Post-operative: 6 days |
| Bjoro (2008) | CAM | 34.3 | Five | Post-operative: 4 days |
| Bowman (1997) | DSM-III | 34.9 | Ten | Post-operative: 5 days |
| Brown (2016) | CAM | 40.5 | Four | Daily |
| Contín (2005) | DSM-III | 22 | Three | Pre-surgery, Post surgery: day 2, day 7 |
| Duprey (2022) | CAM | 23.9 | Multiple | Out of total cohort during hospitalisation |
| Feast (2018) | CAM | 11.4 | Multiple | Prevalence at first assessment |
| Jain (2011) | CAM | 28.3 | Unclear | Prevalence of total sample |
| Johansson (2013) | Neecham | 30.6 | Four | Pre-op, Day 1 post, day 3 post, day before discharge Prevalence figure from pre-op data. |
| Kosar (2014) | CAM | 23.1 | Multiple | Total prevalence following surgery |
| Kubota (2018) | DST/CAM | 9.5 | Unclear | Total prevalence |
| Leung 2009 | CAM | 55 | Three | Pre-operative; post-op day 1 and 2. Prevalence reported overall delirium presence. |
| Leung (2013) | CAM | 40.2 | Three | Prevalence on day 1 or 2 post-operative |
| Lewis (2017) | DSM-V | 19.2 | Once | Total prevalence |
| Li (2019) | CAM-ICU | 28.8 | Four | Total incidence |
| Liang (2014) | CAM | 9.1 | Daily | Postoperative delirium |
| Lin (2016) | NuDesc | 2.2 | Daily | Post-operative: 3 days.  Prevalence is total out of study population. |
| Liu (2022) | CAM | 19.6 | Six | Postoperative: 3 days, twice daily. |
| Lynch (1998) | CAM | 9.4 | Three | Post operative; 3 days |
| Matsuo (2017) | CAM | 16.9 | Three | Three days during intervention |
| Morrison (2003) | CAM | 16.1 | Daily | <48 hours of submission until discharge; prevalence of total cases of delirium |
| Narayanan (2022) | Short-CAM | 4 | Six | Postoperative: 3 days, twice daily. |
| Oh (2008) | CAM | 21.4 | Once | Postoperative |
| Roche-Albero (2021) | CAM | 45.1 | Five | Pre-operative, postoperative days 1 to 4.  Prevalence of delirium outside of the total population. |
| Salottolo (2022) | CAM | 5.4 | Multiple | Throughout admission. Incidence of delirium from arrival through 48 hours post op. |
| Sieber (2011) | CAM | 25.4 | Twice | Pre-operative and day 2 post-operative. Total population prevalence. |
| Susano (2019) | DSM-IV/ ICD discharge codes | 17.8 | Once | Postoperative. |
| Vaurio (2006) | CAM | 47.7 | Three | Pre-op, Postoperative: 2 days. |
| Xue (2016) | CAM | 7.8 | unclear | Evaluated post-operatively…...for a week and at the onset of confusion related symptoms |

CAM: Confusion Assessment Method; DSM: Diagnostic and Statistical Manual of Mental Disorders; ICD: International Classification of Disease; NuDesc: Nursing Delirium Screening Scale: DST: Delirium Screening Tool.

**Supplementary Data 4: Interpretation of a Standard Median Difference (SMD)**

The standardised mean differences (SMD) were calculated using Cohen's method. The standardisation was needed because pain instruments used in each study were on different scales. Thus, a summary measure (i.e., from meta-analysis) calculated with just raw scores would have reflected differences with extra noise (from using different metrics) that would have been hard -or even impossible- to interpret the standardisation approach solves the problem of unequal pain scales, but it requires some explanation. The overall SMD=0.36 (95%CI: 0.18-0.55) in Figure 3 means that the mean pain from the delirium group is '36% of one standard deviation' higher than the mean pain from the non-delirium group.

Note that a standardised measure is assumed to be normally distributed with mean=0 and standard deviation=1, so the mean difference between pain in the delirium group and pain in the non-delirium group is '0.36 standard deviations' as in the following figure:


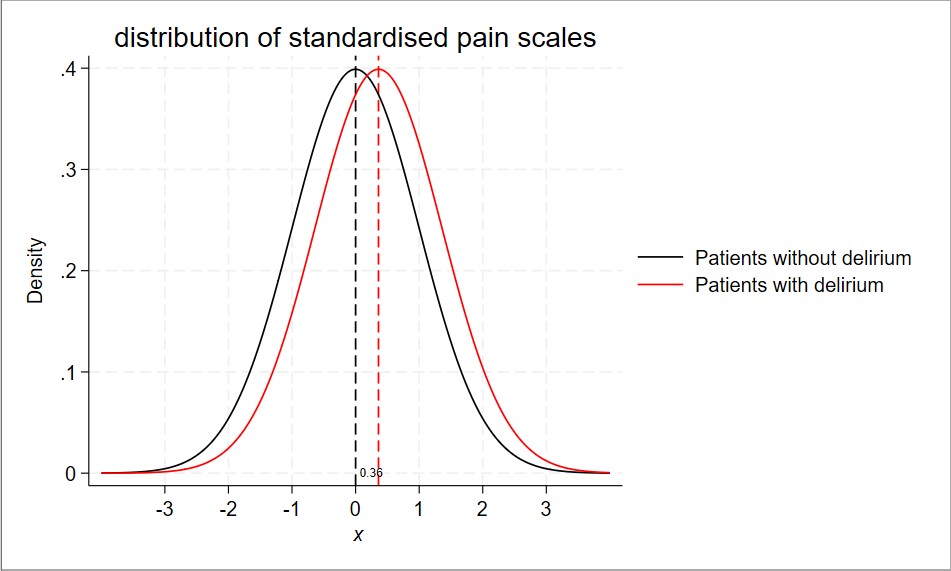


**Supplementary Data 5 : Publication bias funnel plots**

**Figure 1 : Funnel plot for subgroup meta-analysis, which summarises Odds Ratios (using categorical pain data; Figure 2 in the review article)**


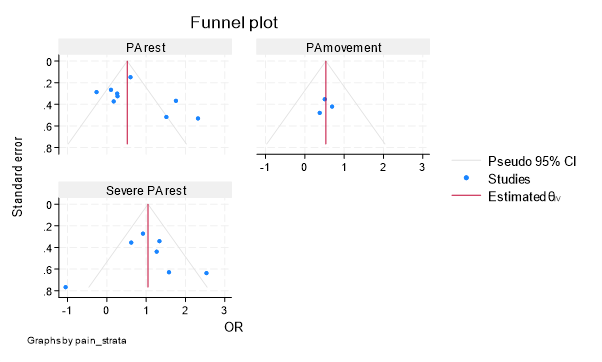


**Figure 2: Funnel plot for meta-analysis summarising Standardised Mean Differences (using continuous pain data; Figure 3 in the review article)**


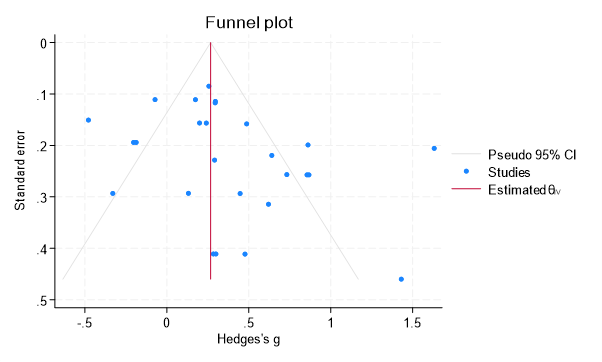


**Supplementary Data 6: Data from controlled analyses**

| **Study** | **Pain type analysed** | **N** | **Measure of effect**  **(95% CI)** | **Factors controlled for** |
| --- | --- | --- | --- | --- |
| Morrison (2003) | Severe pain at rest (VAS 1-5: severe pain = score of 4 or 5) prior to delirium | 242 | RR 9  (1.8-45.2) | Age, functional independence measure, RAND comorbidity score, heart failure on admission, parenteral morphine sulfate equivalents, received meperidine, increased opioid dose after severe pain. |
| Oh  (2008) | Severe pain at rest (VAS 1-10:>6.8) | 224 | OR 1.99  (1.45-4.16) | Previous dementia or delirium, abnormal pre-operative glucose, pre-existent diabetes, local or regional anaesthesia, surgery duration > 3.2 hours, recovery room stay >90minutes, analgesic usage |
| Vaurio  (2006) | Severe pain at rest (VAS ≥ 5) | 302 | OR 3.72  (1.54–8.96) | Age ≥70 years, moderate pain at rest (VAS 1-4 vs.0), increase pain at rest baseline-postoperative day one, neuraxial vs. patient controlled analgesia for postoperative pain control, oral narcotics vs. patient-controlled IV analgesia for postoperative pain control |

# Supplementary Data 7: Data not included in the meta-analysis

Kosar *et al* reported categorical data of pain (mild, moderate, severe) for the participants’ current pain, average pain, and worst pain. They reported the number of delirious cases per group. The authors report that delirium increased with pain severity, but there was a statistically significant risk in the severe current pain group.

| **Worst pain** |  | **total** | **number delirium** | **RR** |
| --- | --- | --- | --- | --- |
|  | mild | 71 | 13 | ref |
|  | moderate | 104 | 21 | 1.1 (0.6 - 2.0) |
|  | severe | 284 | 72 | 1.3 (0.8 - 2.2) |
| average pain |  |  |  |  |
|  | mild | 145 | 29 | ref |
|  | moderate | 205 | 44 | 1.0 (0.7 - 1.6) |
|  | severe | 105 | 33 | 1.4 (0.9 - 2.3) |
| Current pain |  |  |  |  |
|  | mild | 331 | 71 | ref |
|  | moderate | 85 | 16 | 0.9 (0.5 - 1.4) |
|  | severe | 43 | 19 | 2.0 (1.4 - 3.0) |

Narayanan *et al* collected the maximum pain score reported in a 24-hour window, over three days. On each day, they also reported the prevalence of dementia. They reported no significant difference between pain and delirium.

| Pain score (max/24 hr) | Day 1 | | Day 2 | | Day 3 | |
| --- | --- | --- | --- | --- | --- | --- |
|  | No delirium | Delirium | No delirium | Delirium | No delirium | Delirium |
|  | N=48 (96%) | N=2 (4%) | N=41 (82%) | N=9 (9%) | N=39 (78%) | N=11 (22%) |
| 0 | - | - | - | - | - | - |
| 1-3 | 15 (31.25) | - | 32 (78.05) | 4 (44.44) | 36 (92.31) | 11 (100) |
| 4-7 | 33(68.75) | 2 (100) | 9 (21.95) | 5 (55.56) | 3 (7.69) | - |
| 8-10 | - | - | - | - | - | - |
